# Preclinical Characterization of the Anti-Leukemia Activity of the CD33/CD16a/NKG2D Immune-Modulating TriNKET^®^ CC-96191

**DOI:** 10.3390/cancers16050877

**Published:** 2024-02-22

**Authors:** Margaret C. Lunn-Halbert, George S. Laszlo, Sarah Erraiss, Mark T. Orr, Heidi K. Jessup, Heather J. Thomas, Henry Chan, Mahan A. Jahromi, Jonathan Lloyd, Ann F. Cheung, Gregory P. Chang, Tanmay Dichwalkar, Daniel Fallon, Asya Grinberg, Eduardo Rodríguez-Arbolí, Sheryl Y. T. Lim, Allie R. Kehret, Jenny Huo, Frances M. Cole, Samuel C. Scharffenberger, Roland B. Walter

**Affiliations:** 1 Translational Science and Therapeutics Division, Fred Hutchinson Cancer Center, Seattle, WA 98109, USA; 2 Immuno-Oncology Cellular Therapy Thematic Research Center, Bristol Myers Squibb, Seattle, WA 98109, USA; 3 Bristol Myers Squibb, San Diego, CA 92121, USA; 4 Dragonfly Therapeutics, Waltham, MA 02451, USA; 5 Department of Hematology, Hospital Universitario Virgen del Rocío, Instituto de Biomedicina de Sevilla (IBIS/CSIC/CIBERONC), University of Seville, 41013 Seville, Spain; 6 Molecular Medicine and Mechanisms of Disease (M3D) Ph.D. Program, University of Washington, Seattle, WA 98195, USA; 7 Department of Medicine, Division of Hematology and Oncology, University of Washington, Seattle, WA 98195, USA; 8 Department of Laboratory Medicine and Pathology, University of Washington, Seattle, WA 98195, USA

**Keywords:** acute myeloid leukemia (AML), CD33, immunotherapy, natural killer (NK) cell, trispecific antibody

## Abstract

**Simple Summary:**

Current therapies are often insufficiently effective in patients with acute myeloid leukemia (AML), prompting ongoing interest in developing new drugs for this aggressive type of blood cancer. Many efforts focus on drugs that target CD33, a cell surface protein that is widely expressed on AML cells. Gemtuzumab ozogamicin (GO), which consists of a CD33 antibody linked to a small molecule toxin, benefits some AML patients but not many others, leaving room for other CD33-targeted therapeutics. Here, we examined CC-96191, a “TriNKET” protein drug that binds CD33 on AML cells at the same time it binds two different proteins, CD16a and NKG2D, on natural killer (NK) cells, thereby activating these immune cells and engaging them to kill AML cells. In preclinical studies, we found CC-96191 to be broadly active against human AML cells that express CD33. CC-96191 activated NK cells but not T cells; while maximum anti-AML efficacy was similar, soluble cytokine levels were 10- to >100-fold lower than with a protein drug that binds CD33 and CD3 on T cells. Moreover, CC-96191 eliminated AML cells but not normal CD33+ monocytes, suggesting selectivity toward leukemic cells. Together, these findings support the clinical exploration of CC-96191 as is currently ongoing.

**Abstract:**

Increasing efforts are focusing on natural killer (NK) cell immunotherapies for AML. Here, we characterized CC-96191, a novel CD33/CD16a/NKG2D immune-modulating TriNKET^®^. CC-96191 simultaneously binds CD33, NKG2D, and CD16a, with NKG2D and CD16a co-engagement increasing the avidity for, and activation of, NK cells. CC-96191 was broadly active against human leukemia cells in a strictly CD33-dependent manner, with maximal efficacy requiring the co-engagement of CD16a and NKG2D. A frequent CD33 single nucleotide polymorphism, R69G, reduced CC-96191 potency but not maximal activity, likely because of reduced CD33 binding. Similarly, the potency, but not the maximal activity, of CC-96191 was reduced by high concentrations of soluble CD33; in contrast, the soluble form of the NKG2D ligand MICA did not impact activity. In the presence of CD33+ AML cells, CC-96191 activated NK cells but not T cells; while maximum anti-AML efficacy was similar, soluble cytokine levels were 10- to >100-fold lower than with a CD33/CD3 bispecific antibody. While CC-96191-mediated cytolysis was not affected by ABC transporter proteins, it was reduced by anti-apoptotic BCL-2 family proteins. Finally, in patient marrow specimens, CC-96191 eliminated AML cells but not normal monocytes, suggesting selectivity of TriNKET-induced cytotoxicity toward neoplastic cells. Together, these findings support the clinical exploration of CC-96191 as in NCT04789655.

## 1. Introduction

Only a minority of patients with acute myeloid leukemia (AML) are currently cured [1,2,3,4,5]. Because the myeloid differentiation antigen, CD33, is expressed on at least a subset of AML blasts in almost all patients and possibly on underlying leukemia stem cells in some [6,7,8,9,10,11], immunotherapies targeting CD33 have long been pursued to improve these outcomes [10,12]. Results with two unconjugated CD33 monoclonal antibodies (mAbs), lintuzumab (HuM195, SGN-33) and BI-836858, have been disappointing [13,14,15], but reduced relapses and longer survival with the CD33 antibody-drug conjugate gemtuzumab ozogamicin (GO), when added to intensive chemotherapy, validate CD33 as a drug target [11,16]. However, many patients do not benefit from GO, partly because of ATP-binding cassette (ABC) transporter protein activity in AML cells [11], prompting interest in more effective CD33-directed therapies.

The success of T cell-engaging bispecific antibodies (BsAbs) in lymphoid malignancies has raised enthusiasm for similar therapeutics in AML-targeting CD33. Several CD33/CD3 BsAbs have entered clinical testing, with initial results indicating some efficacy [17,18,19,20,21]. However, toxicities from activated T cells cause significant challenges for their use, providing a strong rationale to explore drugs engaging other immune cells such as natural killer (NK) cells that might elicit potent anti-AML activity more safely due to their production of reduced levels of cytokines compared with T cells and their ability to discriminate healthy and malignant cells [22,23,24,25,26]. The latter may result, for example, in less profound myelosuppression when targeting antigens expressed on normal hematopoietic cells such as CD33. CC-96191 is a TriNKET^®^ targeting CD33 on AML cells while activating NK cells through the co-engagement of CD16a (Fc gamma receptor IIIa, FcγRIIIA) and NKG2D. While resting NK cells are activated by CD16a but not NKG2D, NKG2D synergizes with CD16a to activate NK cells more potently [27]. CC-96191 leverages the ability of NK cells to discriminate between healthy and neoplastic CD33+ cells. Here, we characterized the pre-clinical anti-tumor activity of CC-96191 using a variety of shorter-term in vitro assays to define the principles underlying the drug’s cytolytic effects, and contrasted the results to those obtained with a potent CD33/CD3 BsAb.

## 2. Materials and Methods

### 2.1. Therapeutics

CC-96191, CC-96191^ΔNKG2D^, CC-96191^ΔCD33^, CC-96191^FC-SILENT^, CD33 mAb I07, and a CD33/CD3 BsAb were provided by Celgene/Bristol Myers Squibb (Seattle, WA, USA). Lintuzumab was obtained from Creative Biolabs (Shirley, NY, USA).

### 2.2. Lentiviral Expression Vectors

IRES/EGFP cassette-containing vectors encoding full-length CD33 (CD33^FL^), a CD33 variant lacking the membrane-distal V-set domain (CD33^∆E2^), CD33^A14V^, CD33^R69G^, CD33^G304R^, ABCB1 (P-glycoprotein), ABCG2 (breast cancer resistance protein [BCRP]), BCL-2, BCL-xL, or MCL-1 have been described [28,29,30]. Similar vectors encoding CD33^S128N^ or CD16a^158V^ were generated via standard PCR cloning procedures. All constructs were confirmed by Sanger sequencing.

### 2.3. Surface Plasmon Resonance (SPR)

Simultaneous binding of CD16a and NKG2D and co-engagement between CD33 and NKG2D of CC-96191 or trastuzumab were studied with a Biacore 8K instrument and associated analysis software (Cytiva, Pittsburgh, PA, USA) using Series S Sensor CM5 chips as well as human CD16a (158F, ACRO Biosystems, Newark, DE, USA), human NKG2D (SINO Biological, Wayne, PA, USA), human NKG2D-mouse IgG2a Fc (Dragonfly Therapeutics, Waltham, MA, USA), and recombinant human CD33-His (ACRO Biosystems, Newark, DE, USA).

### 2.4. Peripheral Blood Mononuclear Cells and Primary Human NK Cells

Freshly isolated PBMC from healthy adult donors were obtained from Bloodworks Northwest (Seattle, WA, USA). NK cells were isolated using a kit and manufacturer’s instructions (Stemcell Technologies, Kent, WA, USA). Cell pellets were resuspended in pre-warmed RPMI-1640 containing 10% fetal bovine serum (FBS), 1× GlutaMax, and 50 μM 2-ME. Isolated NK cells were rested overnight at 37 °C in 5% CO_2_.

### 2.5. Secreted Cytokine Measurements

Culture supernatants were analyzed utilizing a human proinflammatory 9-plex kit to quantify GM-CSF, IFN-γ, IL-10, IL-12p70, IL-1β, IL-2, IL-6, IL-8, and TNF-α (Meso Scale Discovery, Rockville, MD, USA).

### 2.6. Human NK and Acute Leukemia Cell Lines

Parental KHYG-1 cells (obtained from DSMZ [German Collection of Microorganisms and Cell Cultures GmbH], Braunschweig, Germany) and KHYG-1 cells transduced with CD16a^158F^ (KHYG-1^CD16a^) were cultured in RPMI-1640 with 10% FBS and 100 U/mL recombinant IL-2 (Peprotech, Cranbury, NJ, USA). HL-60, K562, KG-1, ML-1, MOLM-13, OCI-AML3, TF-1, and REH cells were cultured as described [29,31]. THP-1 and EOL-1 cells were cultured in RPMI-1640 with 10% FBS. MV4;11 cells were maintained in IMDM with 10% FBS, 5 ng/mL of GM-CSF, and insulin–transferrin–selenium supplement (Thermo Fisher Scientific, Waltham, MA, USA). Single cell clones of CD33-deficient AML cell sublines were generated via clustered regularly interspaced short palindromic repeat (CRISPR)/Cas9-editing as described [29,31]. Lentivirally transduced sublines were generated at multiplicities of infection of 1–25; EGFP-positive cells were isolated by FACS and re-cultured for further analysis/use.

### 2.7. Quantification of CD33 Expression

CD33 expression was quantified using P67.6-phycoerythrin and QuantiBRITE PE beads (both BD Biosciences, Franklin Lakes, NJ, USA). To identify non-viable cells, samples were stained with 4′,6-diamidino-2-phenylindole (DAPI). DAPI-negative cells were analyzed using FlowJo (version 10; BD Biosciences).

### 2.8. Assessment of Binding of CC-96191 to CD33^FL^ and CD33^∆E2^

Parental REH cells and REH cells transduced with CD33^FL^ or CD33^∆E2^ were labeled with CC-96191 or a chimeric version of 9G2 (C2-set domain-directed CD33 mAb [31]), followed by incubation with an F(ab’)2-goat anti-human IgG Fc secondary antibody (Thermo Fisher Scientific), staining with DAPI, and flow cytometric analysis.

### 2.9. Quantification of Drug-Induced Cytotoxicity

For experiments with KHYG-1 cells, 5 × 10^3^ target cells/well were incubated with KHYG-1 cells with/without CC-96191 or other TriNKET molecules. After 2 days, cell numbers and NK cell- as well as drug-induced cytotoxicity, using DAPI to detect non-viable cells, were determined flow cytometrically. For experiments with other cytotoxic agents, AML cells were incubated with/without GO (Pfizer, New York, NY, USA) or mitoxantrone (Sigma-Aldrich, St. Louis, MO, USA) for 3 days, after which, cell numbers and drug-induced cytotoxicity were quantified. For experiments with primary human NK cells, target cells were labeled with BATDA (PerkinElmer, Shelton, CT, USA) and then incubated with NK cells with/without TriNKET molecules for 2–3 h before time-resolved fluorescence (TRF) analysis. For cytotoxicity with primary PBMCs, EOL-1 cells were labeled with CellTrace Violet (Thermo Fisher) before co-culturing with PBMCs and either CC-96191, lintuzumab, or the CD33/CD3 BsAb, and, in some experiments, recombinant soluble CD33-His (amino acids 18–259; Creative Biomart, Shirley, NY, USA) or recombinant MICA Fc (R&D Systems, Minneapolis, MN, USA). After 24 h, live EOL-1 cells were enumerated by flow cytometry using CountBright™ Absolute Counting Beads.

### 2.10. Frequency of CD33 SNPs

Global frequency estimates from the Allele Frequency Aggregator (ALFA) project (https://www.ncbi.nlm.nih.gov/snp/ (accessed on 1 November 2023)) were used to estimate the frequency of CD33 SNPs.

### 2.11. Primary AML Aspirates

The pharmacoscopy high-content imaging platform was used to quantify the effect of CC-96191 in primary AML patient samples [32,33,34]. Bone marrow cells were purified over a Ficoll density gradient and resuspended in RPMI-1640 containing 10% FBS and 0.1% penicillin/streptomycin. Then, 1–2 × 10^4^ cells/well were plated in 384-well PerkinElmer Cell Carrier Ultra plates with/without CC-96191 or lintuzumab. After fixation/permeabilization, resulting monolayers stained with mAbs against CD33, CD14, CD16, CD56, CD34, CD117, CD69, CD54, and CD3 with DAPI. Imaging of the primary cell monolayer was performed using PerkinElmer CLS spinning disk automated confocal microscopes, with non-overlapping, sequential, fluorescent channel imaging.

### 2.12. Statistical Considerations

Drug specific cytotoxicity is presented as % cytotoxicity = 100 × (1-live target cells treated/live target cells control). Results are presented as mean ± standard error of the mean (SEM) using GraphPad Prism (version 10.1.0; La Jolla, CA, USA).

## 3. Results

### 3.1. CC-96191 Simultaneously Binds CD33, NKG2D, and CD16a with Greater Strength Than Single Binding to NKG2D or CD16a

**Figure 1:**
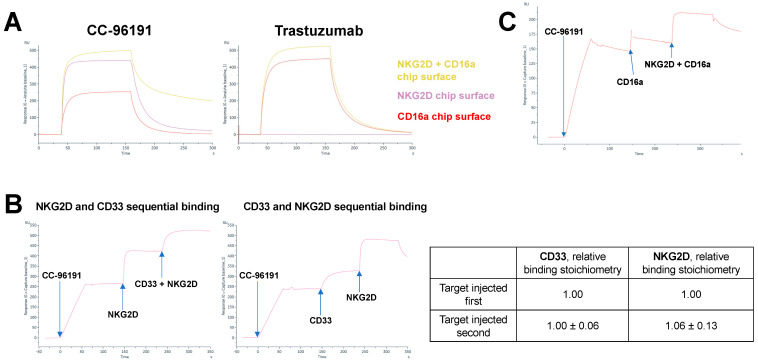
Binding of CC-96191 to CD33, NKG2D, and CD16a. (**A**) SPR analysis of the binding of CC-96191 or trastuzumab (*analyte*) to human CD16a (158F), human NKG2D, or combined CD16a and NKG2D. NKG2D alone (12.3 μg/mL), CD16a alone (9 μg/mL), or the mixture of NKG2D and CD16a at the same concentrations each were amine-coupled to the surface of a CM5 Series S Biacore chip. A total of 1.8 μM CC-96191 or trastuzumab were injected for 150 s at 10 μL/min. Dissociation phase was observed for 180 s when regeneration was not needed and 1800 s when natural regeneration of the surface (almost complete dissociation of analyte) was needed between the cycles at the same flow rate. (**B**) SPR analysis of the sequential binding of saturating concentrations of CD33 (100 nM) and NKG2D (7000 nM) in both orientations across the surface of a Biacore CM5 chip onto which CC-96191 was Fc-captured, with calculation of relative binding stoichiometries. Note: A mixed solution of CD33 and NKG2D was included in the second injection of the first sensorgram to maintain a saturating concentration of NKG2D due to the fast dissociation of NKG2D from CC-96191. (**C**) SPR analysis of the sequential binding of mFc-NKG2D (7000 nM) and CD16a (3000 nM) when injected in sequential order across CC-96191 when captured via CD33-His that was amine coupled to a Biacore CM5 chip.

CC-96191 is a heterodimeric tri-specific antibody composed of a binder derived from a CD33 mAb (mAb I07), a binder derived from a NKG2D mAb, and the constant region of human IgG1 for the co-engagement of CD16a. To evaluate whether the co-engagement of CD16a and NKG2D enhances binding strength to NK cells, we performed SPR studies in which CC-96191 was injected over amine-coupled CD16a (158F), NKG2D, or both CD16a and NKG2D. Compared with CD16a or NKG2D alone, binding strength was increased with simultaneous binding to CD16a and NKG2D with CC-96191 but not trastuzumab, a humanized IgG1 mAb recognizing epidermal growth factor receptor 2 (HER2; Figure 1A). An analysis of binding stoichiometries in experiments in which CC-96191 was immobilized showed that bound CD33 did not interfere with NKG2D binding and vice versa (Figure 1B). Finally, as shown in Figure 1C, CC-96191 was able to concurrently engage CD33, NKG2D, and CD16a in SPR experiments in which NKG2D and CD16a were sequentially injected across CC-96191 when immobilized on the chip surface via CD33. Together, these studies showed that CC-96191 simultaneously binds CD33, NKG2D, and CD16a, and suggested that the co-engagement of NKG2D and CD16a increases the strength of the interaction between the TriNKET and NK cells.

### 3.2. Co-engagement of CD16a, NKG2D, and CD33 Is Required for Optimal Cytolytic Activity of CC-96191 against CD33+ AML Cells

Primary NK cells were used to confirm potent lysis of CD33+ target cells with CC-96191. AML cells often express high levels of the high and intermediate affinity Fc-receptors (FcRs), FcγRI (CD64) and FcγRII (CD32). For example, THP-1 is a human AML cell line known to express high levels of FcγRI and FcγRII, and the addition of THP-1 cells to rituximab-coated Daudi cells has been shown to thwart the therapeutic effect of rituximab [35]. In studies using CD64- MOLM-13 or CD64+ THP-1 AML cell lines as target cells, CC-96191 led to greater antibody-dependent cellular cytotoxicity (ADCC) than the parental CD33 mAb I07, the CD33 mAb lintuzumab, or an afucosylated version of CD33 mAb I07 (Supplemental Figure S1).

**Figure 2:**
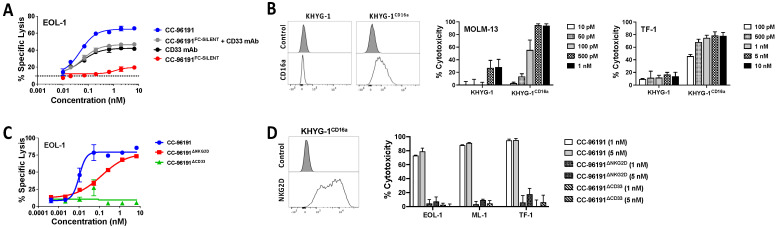
Impact of individual binding arms on CC-96191-induced cytotoxicity. (**A**,**C**) NK cells from healthy donors were incubated for 2 to 3 h with BATDA-labeled CD33+ EOL-1 target cells at an effector:target (E:T) cell ratio of 5:1 in the presence or absence of (**A**) CC-96191, CC-96191^FC-SILENT^, CD33 mAb I07, or CC-96191^FC-SILENT^ and CD33 mAb I07 or (**C**) CC-96191, CC-96191^ΔNKG2D^, or CC-96191^ΔCD33^. (**B**) CD33+ MOLM-13 or TF-1 cells were incubated with parental (CD16a-) KYHG-1 or KHYG-1^CD16a^ cells at an E:T cell ratio of 5:1 with various concentrations of CC-96191. After 2 days, cell numbers and the percentage of dead cells were quantified by flow cytometry. Data are presented as mean ± SEM from 3 independent experiments performed in duplicate wells. (**D**) CD33+ target cells (EOL-1, ML-1, TF-1) were incubated with KHYG-1^CD16a^ cells at an E:T cell ratio of 3:1 in the presence or absence of CC-96191, CC-96191^ΔNKG2D^, or CC-96191^ΔCD33^ at 1 or 5 nM as indicated. After 2 days, cell numbers and the percentage of dead cells were quantified by flow cytometry. Data are presented as mean ± SEM from 3 independent experiments performed in duplicate wells.

We next determined the contribution of each binding arm to the cytolytic activity of CC-96191, using two complementary approaches to study the impact of CD16a engagement. In the first, short-term in vitro cytotoxicity assays were performed with CC-96191 and an Fc-mutant variant of CC-96191 to abrogate binding to CD16a (CC-96191^FC-SILENT^). In the presence of primary human NK cells, CC-96191^FC-SILENT^ was substantially less effective against CD33+ EOL-1 (Figure 2A) cells than CC-96191 in mediating ADCC, indicating a pivotal role of CD16a binding for CC-96191 activity. Combining CC-96191^FC-SILENT^ with the parent CD33 mAb I07 did not restore the full cytolytic activity of CC-96191, suggesting that the co-engagement of NKG2D and CD16a on NK cells by the same molecule is important for anti-tumor activity. In the second approach, we took advantage of endogenously CD16a-deficient KHYG-1 cells and a CD16^158V^-transduced subline of this human NK cell line (KHYG-1^CD16a^) and found that CC-96191-induced cytotoxicity was substantially greater with KHYG-1^CD16a^ than parental KHYG-1 cells (Figure 2B and Supplemental Figure S2).

To study the impact of NKG2D and CD33 engagement on the cytolytic activity of CC-96191 against human CD33+ AML cell lines, we generated variants of CC-96191 in which either the NKG2D binding arm or the CD33 binding arm were replaced with irrelevant binding domains. In the presence of healthy donor NK cells, the CD33 dead-arm TriNKET (CC-96191^ΔCD33^) was ineffective against CD33+ EOL-1 cells, whereas the NKG2D dead-arm TriNKET (CC-96191^ΔNKG2D^) showed reduced cytolytic activity compared with CC-96191 (Figure 2C). Likewise, with KHYG-1^CD16a^ cells, CC-96191 caused substantially greater cytolysis of CD33+ EOL-1, ML-1, or TF-1 cells than either the CC-96191^ΔNKG2D^ or the CC-96191^ΔCD33^TriNKET (Figure 2D), again demonstrating NKG2D and CD33 co-engagement is required for maximal cytolytic activity of CC-96191 against CD33+ human AML cell lines.

### 3.3. Effect of CD33 Expression and CD33 SNPs on CC-96191-Induced Cytotoxicity

We used a large panel of parental and CD33-engineered human acute leukemia cells to characterize the in vitro activity of CC-96191 with KHYG-1^CD16a^ cells. As summarized in Table 1, the sensitivity of CD33+ AML cells or CD33-transduced acute lymphoblastic leukemia (ALL) cells to CC-96191 varied substantially. There was a greater susceptibility of CD33^BRIGHT^ cells (REH^CD33FL^) to CC-96191 than CD33^INTERMEDIATE^ (EOL-1, HL-60, MOLM-13, ML-1, MV4;11, TF-1) cells. Very limited to no cytotoxicity was observed in two CD33^DIM^ cells (KG-1, OCI-AML3) with CC-96191 up to a dose of 5 nM. CD33-negative REH cells and sublines of CC-96191-sensitive ML-1 and TF-1 cells in which CD33 was removed via CRISPR/Cas9 were completely resistant to CC-96191 when used up to 5 nM (Table 1), together demonstrating the CD33-specificity of CC-96191-induced cytotoxicity.

**Figure 3:**
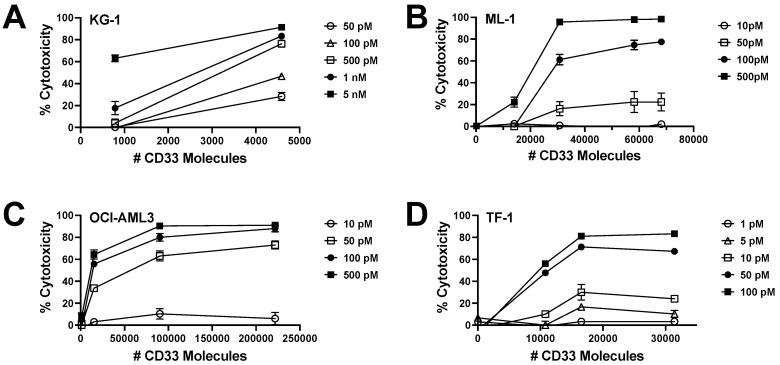
Relationship between CD33 expression and CC-96191-induced cytotoxicity. Sublines of (**A**) KG-1, (**B**) ML-1, (**C**) OCI-AML3, and (**D**) TF-1 cells, genetically engineered to express different levels of CD33 (as determined by QuantiBRITE), were incubated with KHYG-1^CD16a^ cells at an E:T cell ratio of 3:1 in the presence or absence of CC-96191 at various concentrations as indicated. After 2 days, cell numbers and the percentage of dead cells were quantified by flow cytometry. Data are presented as mean ± SEM from 3 independent experiments performed in duplicate wells.

To study whether CD33 expression might be a limiting factor for the anti-tumor activity of CC-96191, sublines of OCI-AML3 and KG-1 cells (both cell lines with low endogenous CD33 expression) were engineered to overexpress wild-type CD33 at increasing levels. Additionally, ML-1 and TF-1 cells were generated with the CRISPR/Cas9-mediated deletion of CD33, and then these cells were used to overexpress CD33^FL^ at various levels. As depicted in Figure 3, a quantitative relationship between CD33 expression and CC-96191-induced cytotoxicity was found at lower CD33 densities in all four cell types, indicating that a low abundance of CD33 can limit the anti-AML activity of CC-96191.

GO and most investigational CD33-directed therapeutics target the membrane-distal V-set domain of CD33 [36]. To test which domain is targeted by CC-96191, we performed SPR analyses using different CD33 proteins and conducted flow cytometric binding with parental (CD33^NEG^) REH cells, and REH cells transduced with either CD33^FL^ or CD33^∆E2^. In these studies, CC-96191 only bound to CD33^FL^ but not CD33^∆E2^, demonstrating that CC-96191 targets the membrane-distal V-set domain of CD33 (Supplemental Figure S3).

**Figure 4:**
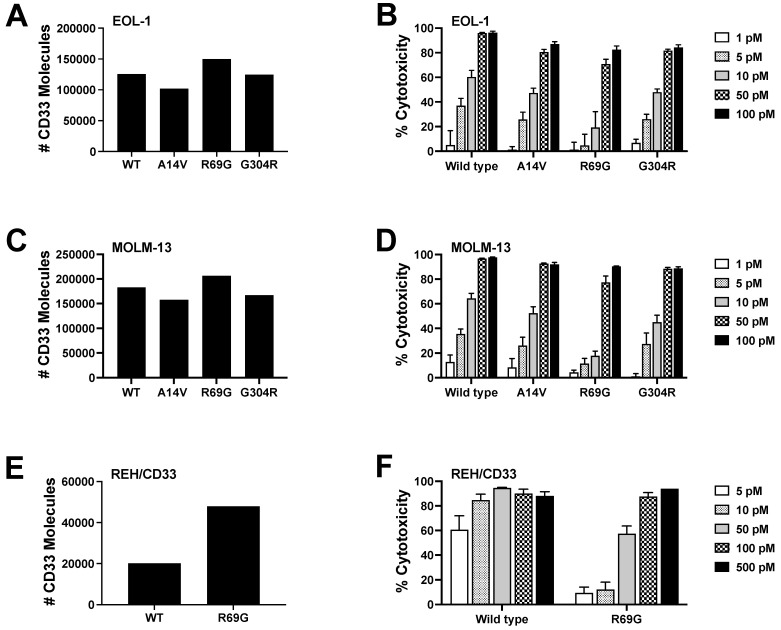
Impact of CD33 SNPs on CC-96191-induced cytotoxicity. (**A**,**B**) MOLM-13 and (**C**,**D**) EOL-1 cells with CRISPR/Cas9-induced deletion of CD33 (CD33KO) and sublines in which either full-length wild-type CD33 (WT) or full-length CD33 A14V, R69G, or G304R were overexpressed via lentivirus to a similar degree were incubated with KHYG-1^CD16a^ cells at an E:T cell ratio of 3:1 in the presence or absence of CC-96191 (1–500 pM) as indicated. (**E**,**F**) REH cells were transfused with either wild-type CD33 (WT) or full-length CD33 R69G and incubated with KHYG-1^CD16a^ cells at an E:T cell ratio of 3:1 in the presence or absence of CC-96191 (1–500 pM) as indicated. After 2 days, cell numbers and the percentage of dead cells were quantified by flow cytometry. Data are presented as mean ± SEM from 3–4 independent experiments performed in duplicate wells.

Three SNPs have been identified within the coding region of CD33 that occur with a minor allele frequency of >10% [37]: A14V (rs12459419, located within the signal peptide of CD33; C>T, with frequency of T allele: 0.26), R69G (rs2455069, located within the V-set domain of CD33; A>G, with frequency of G allele: 0.40), and G304R (rs35112940, located within the intracellular domain of CD33; G>A, with frequency of A allele: 0.19). To investigate the effect of these SNPs on CC-96191-induced cytotoxicity, we used EOL-1 and MOLM-13 cells with CRISPR/Cas9-induced deletion of CD33 and transduced these cells with wild-type CD33^FL^ (CD33^WT^), CD33^A14V^, CD33^R69G^, or CD33^G304R^, and performed in vitro cytotoxicity assays with sublines overexpressing CD33 proteins at similar levels. Compared with target cells expressing CD33^WT^, no appreciable differences in CC-96191-induced cytotoxicity were noted when using target cells expressing either CD33^A14V^ or CD33^G304R^, consistent with the location of these SNPs within the CD33 protein (Figure 4). Conversely, the potency of CC-96191-induced cytotoxicity was reduced with target cells expressing CD33^R69G^ relative to CD33^WT^, but similar levels of ADCC of target cells expressing either CD33^R69G^ or CD33^WT^ were achieved at higher concentrations of CC-96191. Moreover, CC-96191 did not exert any cytotoxicity against cells expressing a rare SNP of CD33 located within the V-set domain, S128N (rs34919259; G>A, with frequency of A allele: 0.0036; Supplemental Figure S4). Since these residues are spatially located near each other on the surface of CD33 (Supplemental Figure S5), these findings suggest that a region within the V-set domain that encompasses R69 and S128 is recognized by the CD33-binding component of CC-96191. Consistently, SPR analyses showed the reduced strength of binding to CD33^R69G^ relative to wild-type CD33, and no quantifiable binding to CD33^S128N^ (Supplemental Figure S6). Together, these data demonstrate the R69G and S128N SNPs’ impact on CC-96191-induced cytotoxicity.

### 3.4. Impact of Soluble MICA and CD33 on CC-96191-Induced Cytotoxicity

Elevated levels of soluble NKG2D ligands, including MHC class I chain-related protein A (MICA), as well as soluble CD33, have been detected in the serum of AML patients [38,39]. The shedding of MICA leads to the reduced NK cell recognition of targets, and the downregulation of the NKG2D receptor [40]. Therefore, we tested whether soluble MICA (shed from the cell surface by proteolytic cleavage, found at levels up to 1–50 ng/mL in the sera of patients with cancer including AML [41,42,43,44], and representing a dominant soluble NKG2D ligand in AML patients [43,44]) and/or CD33 could interfere with CC-96191 cytotoxicity. The addition of 20 ng/mL soluble MICA-Fc (a dimer of MICA monomers expected to bind NKG2D more strongly than monomeric MICA) did not reduce CC-96191-induced cytotoxicity against CD33+ EOL-1, MOLM-13, or THP-1 cells in the presence of primary NK cells (Supplemental Figure S7). Similarly, recombinant soluble CD33 up to 10 ng/mL did not impact CC-96191-induced cytotoxicity against CD33+ EOL-1 cells but at higher concentrations decreased CC-96191 potency (up to 4- to 6-fold at the highest concentrations tested); however, at high doses of CC-96191, lysis reaching 100% was observed even in the presence of high concentrations of soluble CD33 (Supplemental Figure S8).

### 3.5. CC-96191-Induced Cytokine Secretion, Cellular Activation, and Cytolysis by Human NK Cells and PBMCs

**Figure 5:**
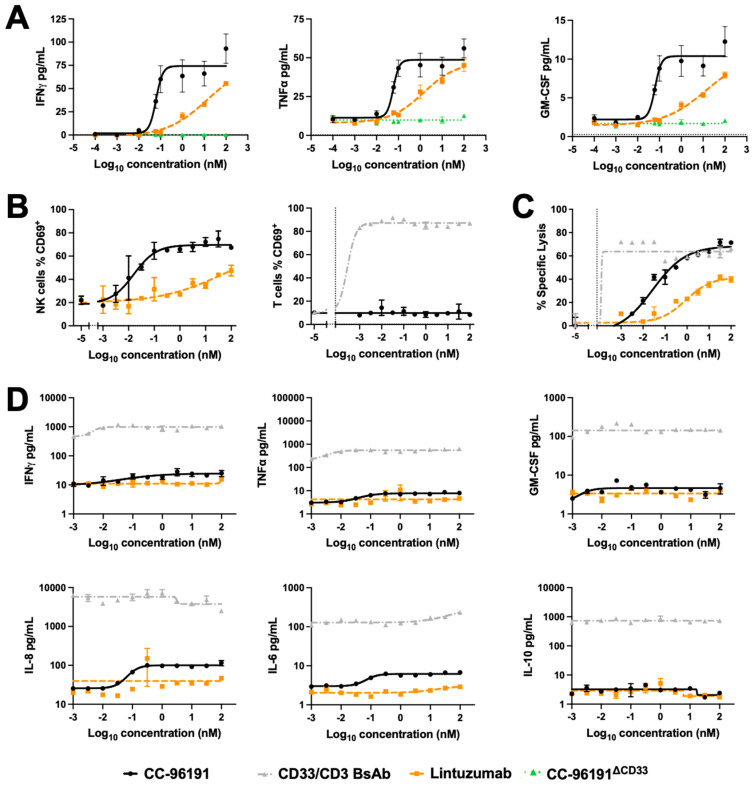
CC-96191 induced cytokine secretion, cellular activation, and cytolysis by human NK cells and PBMCs. (**A**) Healthy donor NK cells were co-cultured with EOL-1 cells for 24 h at an E:T cell ratio of 1:1 in the presence of CC-96191, CC-96191^ΔCD33^, or lintuzumab. Supernatants were collected and analyzed for soluble cytokines. Data are shown as mean +/− S.E.M. from one representative donor of four donors. (**B**–**D**) Healthy donor PBMCs were co-cultured with CTV-labeled EOL-1 cells for 24 h at an E:T cell ratio of 10:1 in the presence of increasing concentrations of CC-96191, lintuzumab, or a CD33/CD3 BsAb before (**B**) CD56+ NK and CD3+ T cell activation were determined by expression of CD69, (**C**) quantification of specific lysis of EOL-1 target cells, and (**D**) analysis of soluble cytokines in culture supernatants. Data are shown as mean +/− S.E.M. from one representative donor of 14 donors.

To test the potential of CC-96191 to induce cytokine secretion by NK cells, we measured cytokine levels in supernatants of NK cells cultured for 24 h with EOL-1 cells. As shown in Figure 5A, CC-96191 more potently induced the secretion of IFN-γ, TNF-α, and GM-CSF than lintuzumab in a strictly CD33-dependent fashion. There was no appreciable production of IL-6, IL-1β, IL-2, IL-12p70, or IL-10 from these co-cultures with CC-96191. We then assessed the CC-96191-mediated activation of PBMC-derived NK and T cells, as determined by the induction of CD69. In the presence of EOL-1 cells, CC-96191 activated NK cells more potently than lintuzumab, whereas it did not activate T cells (Figure 5B). In these PBMC co-cultures, NK cell-activation correlated with the potency of target cell cytolysis, with CC-96191 being substantially more potent than lintuzumab and, at higher doses, reaching the same maximal cytolytic effect as was obtained with a CD33/CD3 BsAb in causing AML cell death (Figure 5C). Compared with the CD33/CD3 BsAb, CC-96191 elicited the secretion of 10-fold to >100-fold lower amounts of TNF-α, IFN-γ, GM-CSF IL-6, IL-8, and IL-10 by PBMCs co-cultured with CD33+ EOL-1 cells (Figure 5D). Thus, at higher concentrations, CC-96191 can generate similar levels of killing of AML cells as a T cell-engaging BsAb without producing high levels of cytokines that might contribute to toxicities.

### 3.6. CC-96191-Induced Selective Destruction of Leukemia Cells in Bone Marrow Samples from Patients with AML

**Figure 6:**
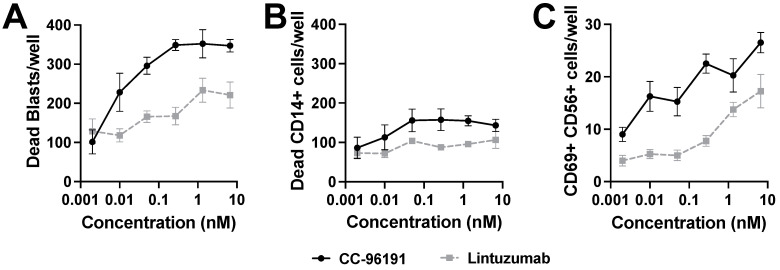
Effects of CC-96191 in AML patient bone marrow. Freshly collected AML bone marrow mononuclear cells were incubated with CC-96191 or lintuzumab for up to 48 h and analyzed by pharmacoscopy. (**A**) Number of non-viable CD34+/CD117+ cells after 48 h, plotted as drug dose response curves. Each point represents the mean value of four technical replicate wells, error bars represent standard error. (**B**) Number of non-viable CD33+/CD14+ cells, plotted as drug dose response curves. Each point represents the mean value of four technical replicate wells, error bars represent standard errors. (**C**) Number of CD69+/CD56+ NK cells, plotted as drug dose response curves. Data are shown as mean ± SEM from representative donor of 5 donors.

We determined the effects of CC-96191 or lintuzumab on freshly collected bone marrow specimens from 10 patients with newly diagnosed (n = 8) or relapsed/refractory (n = 2) CD33+ AML. The pharmacoscopy high-content imaging platform was used to analyze changes in viable and non-viable AML cells and CD14+/CD33+ normal cells as a function of CC-96191 or lintuzumab over time without the addition of exogenous effector cells. At 24 and 48 h, CC-96191 increased the number of non-viable AML cancer cells more potently and to a higher maximal level than lintuzumab (Figure 6A). In contrast, little to no “on-target, off-tumor” killing of normal monocytes was observed (Figure 6B). Of note, this sparing of healthy CD33+/CD14+ cells occurred in the same samples in which CC-96191 efficiently induced AML cell cytolysis. While the pharmacoscopy high-content imaging platform does not permit direct quantification of CD33 levels on cells of interest, comparative in vitro analyses showed that expression levels on normal primary monocytes were similar to those on MOLM-13 cells (Supplemental Figure S9), a cell line chosen for this analysis as a model of AML with intermediate CD33 expression (Table 1). The activity of CC-96191 against the AML cells correlated with an increase in bone marrow NK cell activation, as determined by increased numbers of NK cells expressing CD69, with CC-96191 being more potent than lintuzumab in inducing NK cell activation and achieving a higher maximal number of cells activated (Figure 6C).

### 3.7. Effect of ABC Transporter Protein Expression on CC-96191-Induced Cytotoxicity

Because drug resistance due to ABC transporter proteins such as P-glycoprotein and BCRP is common in AML [45], we generated sublines of three human AML cell lines (EOL-1, MOLM-13, and MV4;11) to overexpress either P-glycoprotein or BCRP and studied their effect on CC-96191-induced cytotoxicity. As expected [28], these sublines were more resistant to GO (P-glycoprotein-overexpressing cells) or mitoxantrone (BCRP-overexpressing cells) relative to their parental counterparts (Supplemental Figure S10). In contrast, neither P-glycoprotein nor BCRP altered target cell killing by CC-96191 (Supplemental Figure S11).

### 3.8. Effect of Anti-Apoptotic BCL-2 Family Proteins on CC-96191-Induced Cytotoxicity

**Figure 7:**
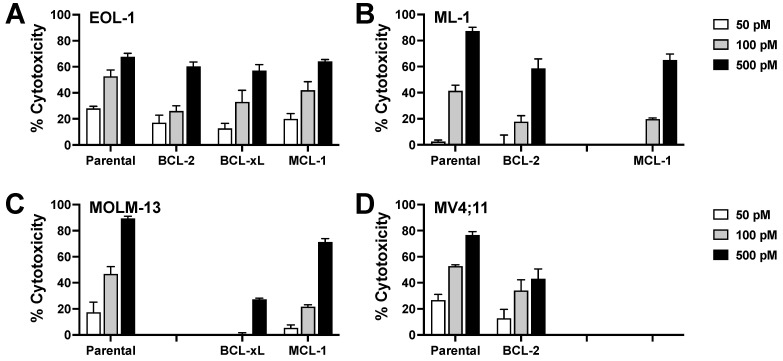
Effect of anti-apoptotic BCL-2 family member proteins on CC-96191-induced cytotoxicity. Sublines of (**A**) EOL-1, (**B**) ML-1, (**C**) MV4-11, and (**D**) MOLM-13 cells were generated to overexpress BCL-2, MCL-1, or BCL-XL via lentivirus-mediated gene transfer. Cells were incubated with KHYG-1^CD16a^ cells at an E:T cell ratio of 3:1 in the presence or absence of CC-96191 at various concentrations as indicated. After 2 days, cell numbers and the percentage of dead cells were quantified by flow cytometry. Data are presented as mean ± SEM from 3 independent experiments with duplicate wells.

As AML often over expresses the anti-apoptotic BCL-2 family proteins, BCL-2, BCL-xL, and MCL-1, we generated sublines of CD33+ EOL-1, ML-1, and MV4;11 cells overexpressing BCL-2, BCL-xL, or MCL-1. As summarized in Figure 7, CC-96191 activity was reduced but not completely abrogated in sublines overexpressing one of these anti-apoptotic BCL-2 family proteins relative to parental cells, indicating that BCL-2 family proteins modulate sensitivity to CC-96191-induced cytotoxicity.

## 4. Discussion

Following haplo-identical hematopoietic cell transplantation (HCT), donor NK cells mediate graft-vs-leukemia benefits, resulting in AML clearance without graft-vs-host disease. Positive outcomes in this setting correlate with an activated NK cell phenotype, an enhanced expression of NK activating receptors, and an expression of activating but not inhibitory receptor ligands on leukemic blasts [8,46,47,48,49,50,51,52,53]. Among others, this observation supports the development of NK cell immunotherapies for AML. While resting NK cells can be activated by CD16a (but not NKG2D) alone, optimal NK cell activation requires the co-engagement of at least two activating NK receptors, such as CD16a and NKG2D [27]. Hence, CC-96191 was designed to enhance the activation of NK cells via engagement of both CD16a and NKG2D within a single therapeutic molecule. Our SPR analyses show that such co-engagement increases binding strength relative to binding to CD16a alone, pointing toward an additional benefit (i.e., the introduction of avidity that manifests as a slower off-rate) of the co-engagement of NK cell receptors with this therapeutic format.

Using genetically defined in vitro models of human AML, primary human AML cells, and various TriNKET molecules, we investigated cellular determinants for the anti-leukemic activity of CC-96191. The results presented herein show that CC-96191 exerts substantial cytolytic activity against a broad panel of human CD33+ AML cells that, unlike the anti-AML activity of GO, is not impacted by ABC transporter proteins. This anti-tumor efficacy was strictly dependent on the presence of CD33. Maximal drug activity required the co-engagement of NKG2D and CD16a and was greater than the ADCC induced by anti-CD33 mAbs, including an ADCC-enhanced afucosylated anti-CD33 mAb—providing credence for the requirement for co-engagement of two activating NK cell receptors for optimized cellular activation and NK cell-mediated cytotoxicity.

Our in vitro experiments indicate that a low abundance of CD33 can limit CC-96191 efficacy, an observation similar to that made previously with GO [11]. This relationship between CD33 expression and CC-96191 activity is important because CD33 expressed on AML cells varies over 2-log-fold across individual patients, and often varies significantly within any given patient but, overall, is relatively limited, in particular on immunophenotypically immature subsets of AML cells [10,11].

Our binding studies confirm CC-96191 recognizes the V-set domain of CD33. Our experiments establish the important impact of CD33 SNPs located within the V-set domain of CD33 for CC-96191-induced cytotoxicity. Specifically, G69 substantially reduced drug efficacy at intermediate doses, and N128 completely abrogated it. Given that these residues are spatially close to each other, our data suggest that CC-96191 binds an epitope that either involves, or is near, these two amino acids. Whereas the S128N SNP is rare, the R69G SNP is frequently occurring, with a minor allele frequency of about 40%. Therefore, the impact of R69G on CC-96191 efficacy may be relevant for clinical drug development and testing.

At sCD33 concentrations representing the upper range (25 to 50 ng/mL) of those anticipated to be present in AML patient plasma, a 2- to 3-fold loss of potency was observed. At concentrations more than the anticipated in vivo concentration of sCD33 (100 ng/mL), a 4- to 6-fold loss in potency was observed. Despite this loss of potency at high concentrations (1 to 10 nM) of CC-96191, 100% maximal lysis was achieved even in the presence of 100 ng/mL sCD33. These data suggest that it should be possible to dose through any inhibition of CC-96191 activity due to the binding of sCD33.

When co-cultured with healthy donor peripheral blood mononuclear and CD33+ AML cells, CC-96191 activated NK cells but not T cells, with resulting soluble cytokine levels 10- to >100-fold lower than those resulting from treatment with a CD33/CD3 BsAb. That higher concentrations of CC-96191 exert cytotoxic effects on CD33+ AML cells that are similar in magnitude to those induced by a potent CD33/CD3 yet result in lower levels of cytokines, suggests the possibility of improved safety with the TriNKET relative to a T cell-engaging BsAb. This safety aspect, as it pertains not only to on-target, on-tumor toxicity but also on-target, off-tumor toxicity, may be particularly important for a myeloid differentiation antigen such as CD33 that is broadly expressed not only on AML cells but also on normal maturing and mature myeloid cells [10,11]. Safety may further be improved by the preferential cytotoxicity of CC-96191 toward neoplastic cells, as indicated by our co-culture experiments with bone marrow specimens from patients with AML.

Improved survival when venetoclax, an oral inhibitor of BCL-2, is added to azacitidine or low-dose cytarabine in adults with newly diagnosed AML [54,55,56] has highlighted the importance of anti-apoptotic BCL-2 family proteins for the survival and chemoresistance of AML cells. Consistent with previous studies showing that the overexpression of such proteins impaired NK cell killing [57], we found BCL-2, BCL-xL, and MCL-1 reduced CC-96191-induced cytotoxicity, suggesting the inhibitors of anti-apoptotic BCL-2 family proteins could serve as rational partners for combination therapies with CC-96191.

While we carefully characterized the preclinical anti-leukemia efficacy of CC-96191 in controlled studies using a large panel of human acute leukemia cell lines, including genetically engineered sublines, primary AML and normal hematopoietic cells as target cells, as well as human NK cell lines and primary human NK cells as immune effector cells, several limitations of this study must be acknowledged. For one, all our experiments with primary human NK cells were limited to shorter-term assays, preventing us from studying the possible effects of CC-96191 on induction of NK cell proliferation and/or NK cell exhaustion. However, our focus on shorter-term assays was purposeful because of the difficulty/inability to maintain the viability of primary NK cells for longer than 1–2 days without supplementation by cytokines such as IL-2 or IL-15; as they can potentiate TriNKET effects, the need for such cytokines would confound any interpretation of our findings. Moreover, because of the lack of cross-reactivity between CC-96191 and murine NK cells, and the difficulties in establishing human NK cells in immunodeficient mice, we focused our studies entirely on in vitro examinations of CC-96191.

## 5. Conclusions

Our findings demonstrate CC-96191 has potent CD33-dependent cytolytic activity in vitro against human AML cells, supporting the drug’s exploration in early phase clinical trials. One such trial (NCT04789655) has opened accrual for adults with relapsed or refractory AML. Relevant for clinical development, our results suggest the value of the CD33 SNP R69G and the activity of anti-apoptotic BCL-2 family proteins (e.g., as assessed via BH3 profiling) as biomarkers that could be useful for patient selection and, in the case of BCL-2 family proteins, the identification of a rational combination therapy.

## Figures and Tables

**Table 1:** Summary of NK cell (KHYG-1^CD16a^)-induced cytotoxicity toward human acute leukemia cell lines in presence/absence of CC-96191.

Cell Line	CD33 Expression	KHYG-1^CD16a^ Cytotoxicity	CC-96191 Specific Cytotoxicity				
			10 pM	50 pM	500 pM	1 nM	5 nM
EOL-1	++	26.0 ± 15.4	*ND*	*ND*	52.7 ± 3.2	62.3 ± 3.8	69.0 ± 4.6
HL-60	++	8.2 ± 3.5	*ND*	2.0 ± 4.2	14.0 ± 3.6	30.7 ± 5.4	46.7 ± 3.5
K562	+	91.4 ± 4.9	*ND*	*ND*	*ND*	*ND*	*ND*
KG-1	+	12.4 ± 1.8	*ND*	*ND*	*No effect*	7.3 ± 8.3	5.0 ± 6.5
ML-1	++	32.6 ± 8.5	*No effect*	*No effect*	34.3 ± 22.2	62.0 ± 15.6	*ND*
ML1^CD33KO^	−	22.8 ± 12.7	*No effect*	*No effect*	*No effect*	*No effect*	*No effect*
MOLM-13	++	25.2 ± 13.8	2.7 ± 1.8	13.3 ± 4.5	94.7 ± 2.4	94.0 ± 3.1	*ND*
MV4;11	++	34.9 ± 9.4	*ND*	*ND*	45.7 ± 17.3	51.0 ± 19.1	53.3 ± 18.7
OCI-AML3	+	22.5 ± 12.9	*No effect*	5.7 ± 1.3	6.0 ± 3.1	1.3 ± 3.4	*ND*
REH	−	25.7 ± 13.2	*No effect*	*No effect*	*No effect*	*ND*	*ND*
REH^CD33FL^	++++	16.7 ± 7.9	21.0 ± 4.4	73.0 ± 7.8	89.0 ± 4.0	*ND*	*ND*
TF-1	+++	28.6 ± 1.6	*ND*	*ND*	68.0 ± 4.6	74.7 ± 4.3	78.3 ± 6.1
TF-1^CD33KO^	−	31.6 ± 6.5	*ND*	*ND*	0.3 ± 3.3	*No effect*	*No effect*

Results are presented as mean ± SEM from 3 independent experiments with duplicate wells. ND, not done; − no expression; +, low expression; ++, intermediate expression; +++, high expression; ++++, very high expression.

## Data Availability

For original data and reagents, please contact the corresponding author (rwalter@fredhutch.org).

## Supplementary Materials

The following supporting information can be downloaded at: https://www.mdpi.com/article/10.3390/cancers16050877/s1, Figure S1: CC-96191-induced cytotoxicity with primary human NK cells; Figure S2: Effect of CD16a expression on CC-96191-induced cytotoxicity; Figure S3: Binding of CC-96191 to human CD33; Figure S4: Effect of CD33 S128N SNP on CC-96191-induced cytotoxicity; Figure S5: Crystal structures of R69 CD33 and G69 CD33; Figure S6: SPR analysis of binding to CD33 R69G and CD33 S128N; Figure S7: Impact of soluble MICA on CC-96191-induced cytotoxicity; Figure S8: Impact of soluble CD33 on CC-96191-induced cytotoxicity; Figure S9: CD33 expression on normal primary monocytes; Figure S10: Effect of P-glycoprotein and BCRP expression on GO- and mitoxantrone-induced cytotoxicity; Figure S11: Effect of P-glycoprotein and BCRP expression on CC-96191-induced cytotoxicity.
