# Supplementary material for: Preclinical Characterization of the Anti-Leukemia Activity of the CD33/CD16a/NKG2D Immune-Modulating TriNKET® CC-96191
Source: Cancers (Basel). 2024 Feb 22;16(5):877. doi: 10.3390/cancers16050877 (PMC10931532; doi:10.3390/cancers16050877)
Supplement: Supplementary file 1 [file cancers-16-00877-s001.zip › cancers-2861438-supplementary.pdf]

SUPPLEMENTAL FIGURES

Supplemental Figure S1

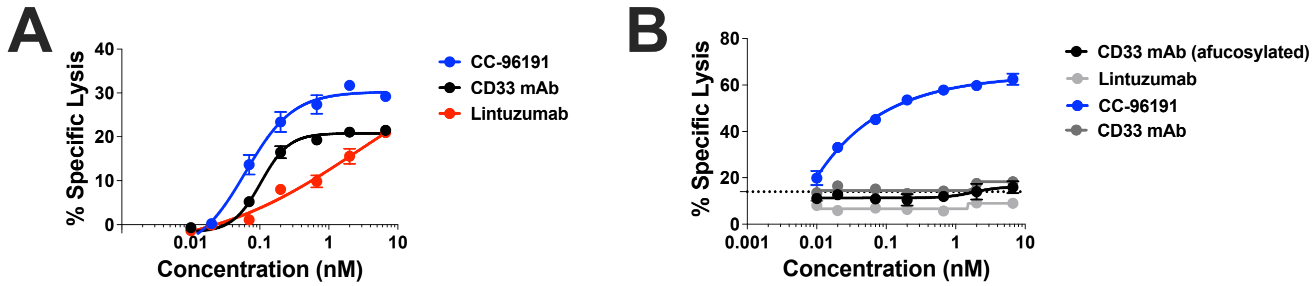

CC-96191-induced cytotoxicity with primary human NK cells. NK cells were isolated from PBMCs from healthy donors, rested overnight, and then incubated for 2 to 3 hours with BATDA labeled **(A)** CD33+ MOLM-13 or **(B)** CD33+ THP-1 target cells at an E:T cell ratio of 5:1 in the presence or absence of CC-96191, lintuzumab, an afucosylated CD33 mAb (clone I07), or standard CD33 mAb (clone I07) as indicated before quantitation of specific lysis.

Supplemental Figure S2

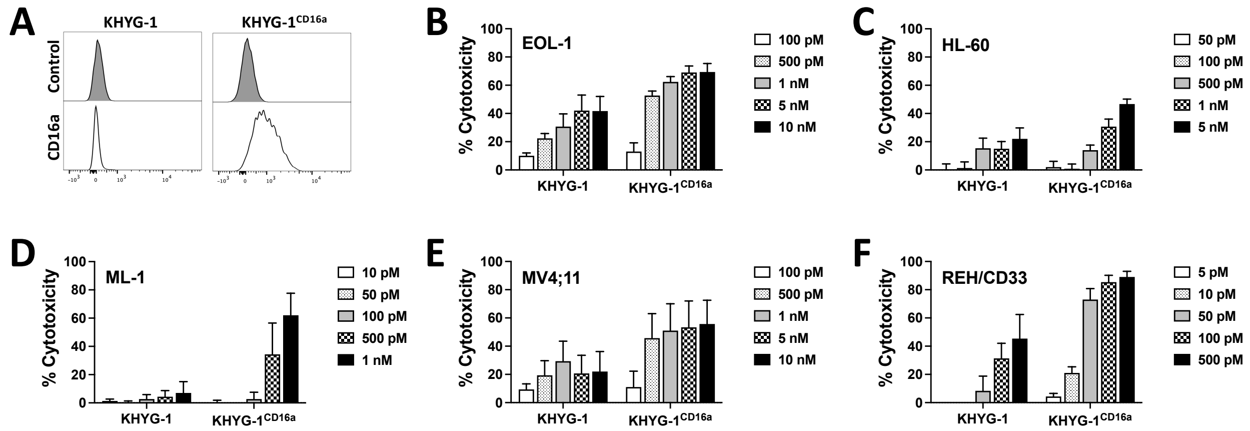

Effect of CD16a expression on CC-96191-induced cytotoxicity. (A) Flow cytometric quantification of CD16a expression on parental KHYG-1 and KHYG-1^{CD16a} cells. 2-day *in vitro* cytotoxicity assays with parental KHYG-1 and KHYG-1^{CD16a} cells and CD33+ human acute leukemia cells (**(B)** EOL-1 cells; **(C)** HL-60 cells; **(D)** ML-1 cells; **(E)** MV4;11 cells; **(F)** REH cells transduced with CD33^{FL}) at an effector:target (E:T) cell ratio of 3:1 in the presence or absence of various doses of CC-96191 as indicated. After 2 days, cell numbers and percentage of dead cells were quantified by flow cytometry. Data are presented as mean ± SEM from 3 independent experiments performed in duplicate wells.

Supplemental Figure S3

A

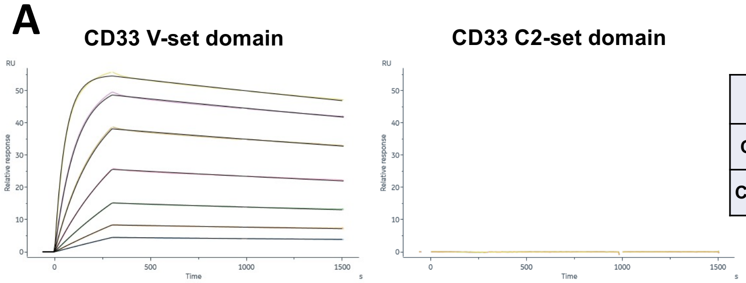

Antigen domain	k_a (1/Ms)	k_d (1/s)	K_D (pM)
CD33 V-set domain	1.8×10^6	1.3×10^{-4}	72.7
CD33 C2-set domain	No binding up to 10 nM		

B

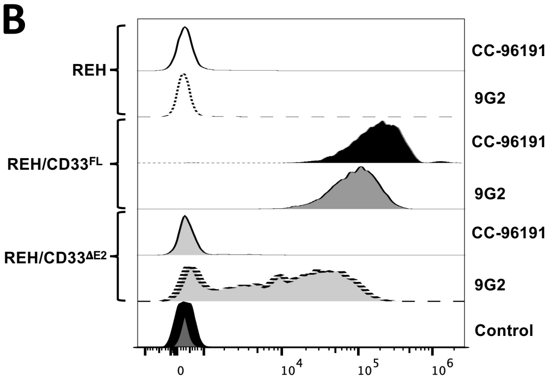

Binding of CC-96191 to human CD33. (A) SPR analysis of binding of CC-96191 to human CD33. CC-96191 was Fc-captured on a Biacore CM5 chip surface. 0.16-10 nM CD33 V-domain or C-domain (Dragonfly Therapeutics, Waltham, MA) were injected across the chip surface in 2-fold dilutions (n=2 per domain) and fit to a 1:1 kinetic fit model, where applicable. **(B)** CC-96191 and 9G2, a mAb recognizing the membrane-proximal C2-set domain of CD33 (Godwin CD, Laszlo GS, Fiorenza S, et al. Targeting the membrane-proximal C2-set domain of CD33 for improved CD33-directed immunotherapy. *Leukemia*. 2021;35(9):2496-2507), were tested flow cytometrically against parental (CD33^{NEG}) REH cells, REH cells transduced with full-length, wild-type CD33 (CD33^{FL}), and REH cells transduced with CD33 variant lacking the exon 2-encoded membrane-distal V-set domain (CD33$^{\Delta E2}$), as indicated. A control without primary mAb was included as well.

Supplemental Figure S4

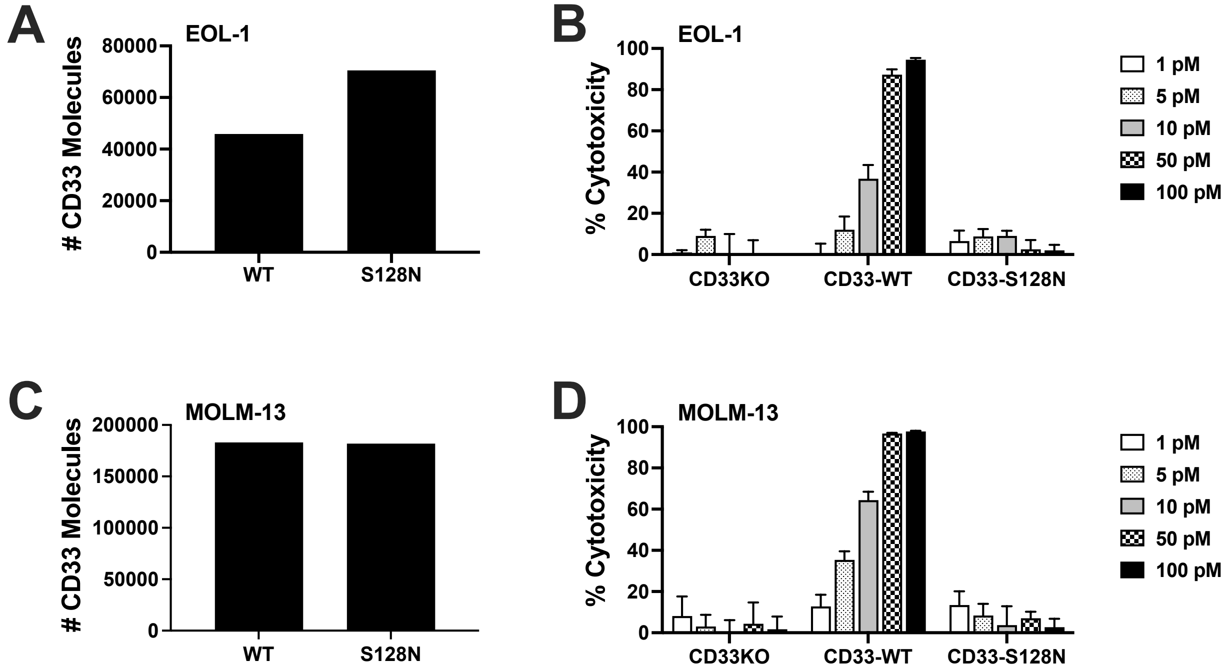

Effect of CD33 S128N SNP on CC-96191-induced cytotoxicity. (A,B) MOLM-13 and **(C,D)** EOL-1 cells with CRISPR/Cas9-induced deletion of CD33 ($CD33^{KO}$) and sublines in which either full-length CD33 ($CD33^{FL}$) or CD33 with a S128N point mutation ($CD33^{S128N}$) were overexpressed via lentivirus to a similar degree were incubated with KHYG-1^{CD16a} cells at an E:T cell ratio of 3:1 in the presence or absence of CC-96191 (1-100 pM) as indicated. After 2 days, cell numbers and the percentage of dead cells were quantified by flow cytometry. Data are presented as mean ± SEM from 3-4 independent experiments performed in duplicate wells.

<u>**Supplemental Figure S5**</u>

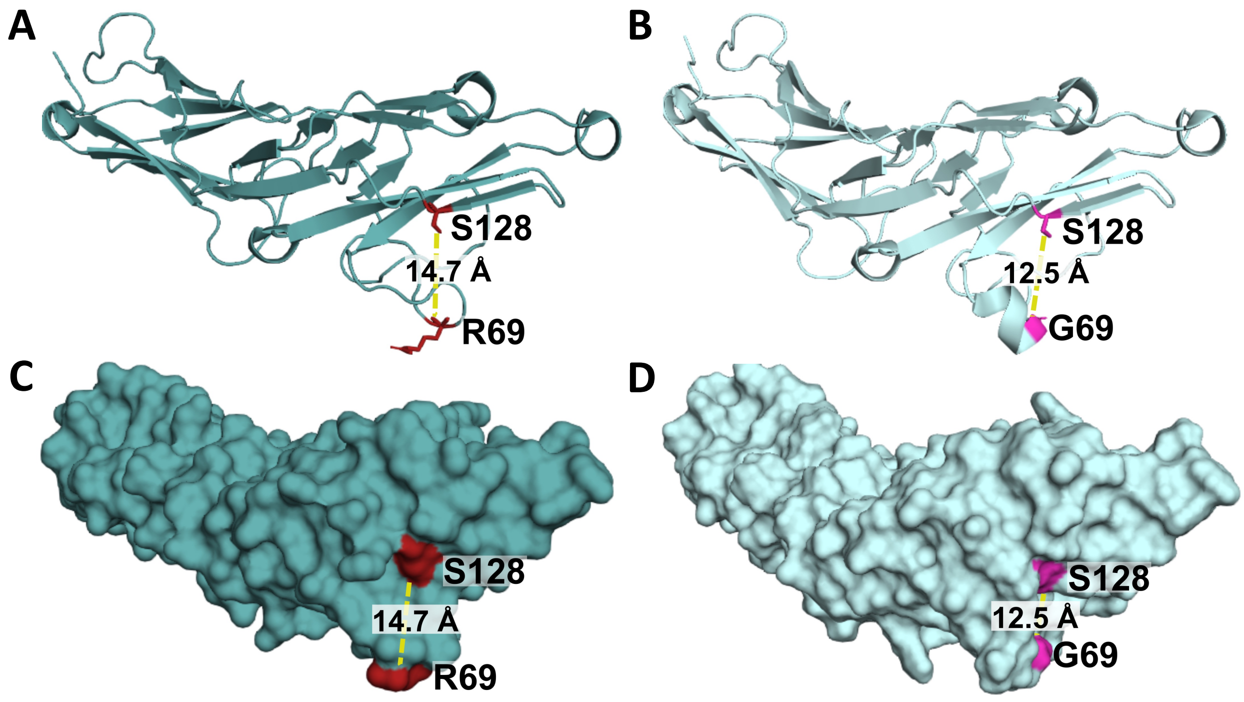

Crystal structures of R69 CD33 and G69 CD33. Crystal structures of the partial extracellular regions of CD33 with either R69 (teal [**A,C**], PDB 7aw6) or G69 SNP (cyan [**B,D**], PDB 5ihb). R69, G69 and S128 are shown in stick format (red and magenta) and distances between the residues are illustrated as dashed lines.

Supplemental Figure S6

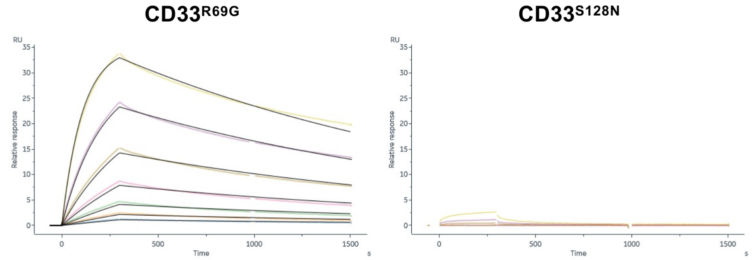

Antigen SNP	k_a (1/Ms)	k_d (1/s)	K_D (pM)
CD33^{R69G}	8.8 x 10^5	4.7 x 10^{-4}	539.9
CD33^{S128N}	No quantifiable binding up to 10 nM		

SPR analysis of binding to CD33 R69G and CD33 S128N. CC-96191 was Fc-captured on a Biacore CM5 chip surface. 0.16-10 nM CD33 R69G or S128N (Dragonfly Therapeutics, Waltham, MA) were injected across the chip surface in 2-fold dilutions (n=2 per antigen) and fit to a 1:1 kinetic fit model, where applicable.

Supplemental Figure S7

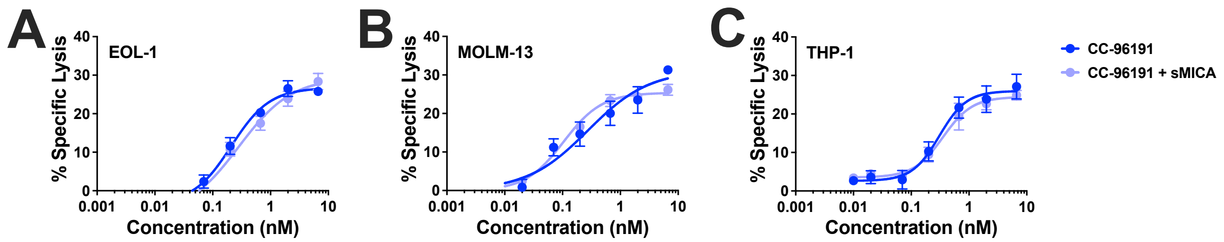

Impact of soluble MICA on CC-96191-induced cytotoxicity. NK cells were isolated from PBMCs from healthy donors, rested overnight, and incubated for 2-3 hours with BATDA-labeled **(A)** CD33+ EOL-1 cells, **(B)** CD33+ MOLM-13 cells, or **(C)** CD33+ THP-1 cells at an E:T cell ratio of 5:1 in the presence of CC-96191 with or without 20 ng/mL of soluble MICA before quantitation of specific lysis.

Supplemental Figure S8

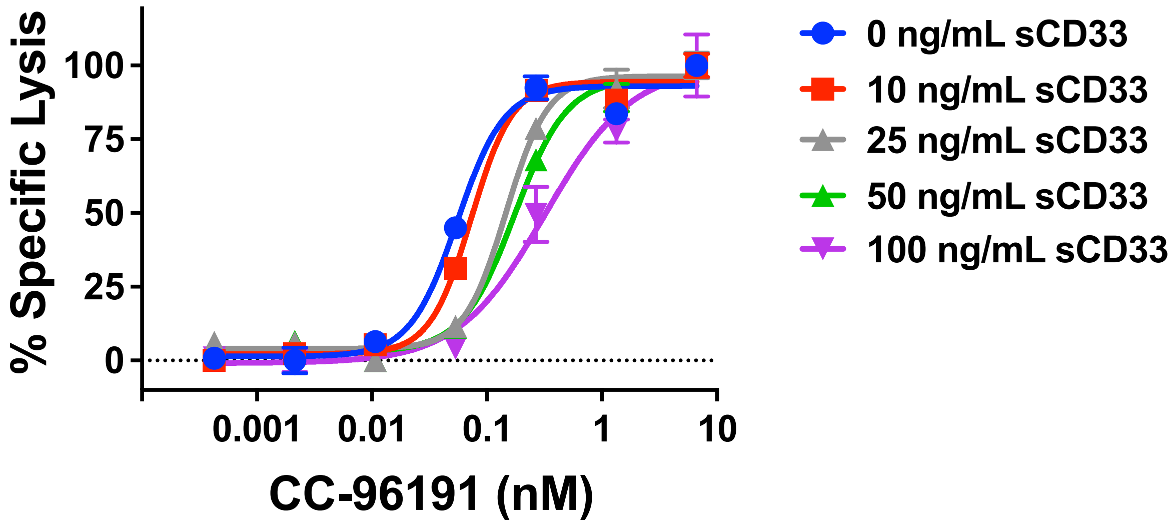

Impact of soluble CD33 on CC-96191-induced cytotoxicity. NK cells were isolated from PBMCs from healthy donors, rested overnight, and incubated for 2-3 hours with BATDA-labeled CD33+ EOL-1 cells at an E:T cell ratio of 5:1 in the presence of CC-96191 with or without 1-100 ng/mL of soluble CD33-His (sCD33) before quantitation of specific lysis. Data are presented as mean ± SEM and are representative of 3 donors with similar results.

Supplemental Figure S9

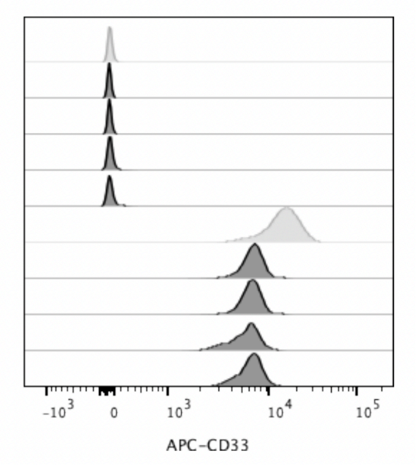

CD33 expression on normal primary monocytes. Histograms of CD33 expression on monocytes from 4 healthy donors (dark gray) and MOLM-13 cell line (light gray). The top five rows are signals from the same samples stained with isotype control antibody. Monocytes and MOLM-13 cells were washed and resuspended in 1x PBS and stained with Fixable Viability dye for 20 min at room temperature. Cells were then centrifuged and resuspended in FACS staining buffer (1x PBS supplemented with 2% FBS) and the following antibodies: APC-CD33, BV421-CD19 and BV-650-CD14. The samples were stained for 15 minutes at room temperature. Subsequently, cells were then centrifuged, resuspended in Fixation Buffer, and incubated on ice for 10 min. Finally, samples were washed with FACS staining buffer twice and resuspended in FACS staining buffer before analysis on a BD LSRFortessa instrument.

Supplemental Figure S10

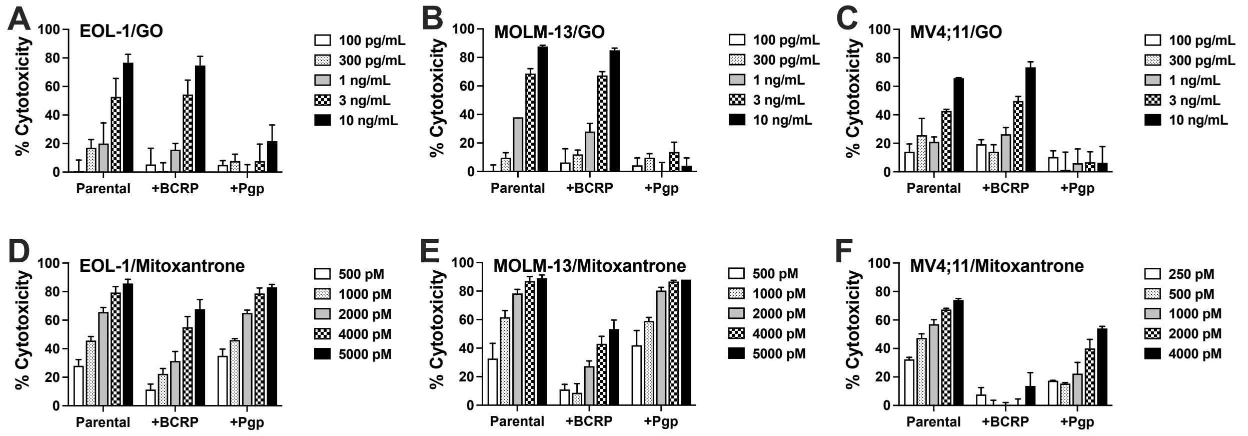

Effect of P-glycoprotein and BCRP expression on GO- and mitoxantrone-induced cytotoxicity. Parental EOL-1, MOLM-13, and MV4;11 cells and sublines transduced with either wild-type P-glycoprotein or BCRP were incubated with increasing concentrations of GO or mitoxantrone. After 2 days, cell numbers and the percentage of dead cells were quantified by flow cytometry. Data are presented as mean ± SEM from 3 independent experiments performed in duplicate wells.

Supplemental Figure S11

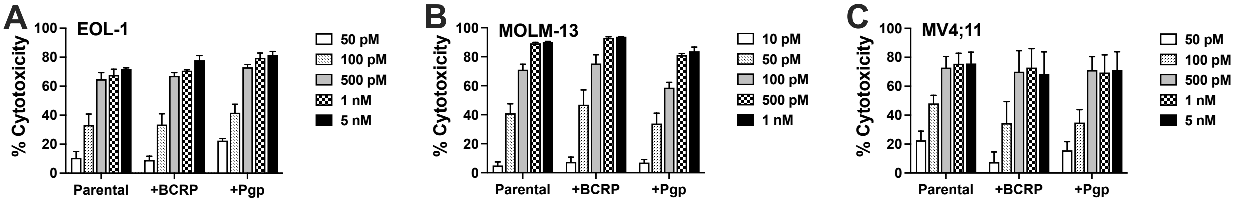

Effect of P-glycoprotein and BCRP expression on CC-96191-induced cytotoxicity. Parental **(A)** EOL-1, **(B)** MOLM-13, and **(C)** MV4;11 cells and sublines transduced with either wild-type P-glycoprotein or BCRP were incubated with KHYG-1^{CD16a} cells at an E:T cell ratio of 3:1 with/without CC-96191. After 2 days, cell numbers and the percentage of dead cells were quantified by flow cytometry. Data are presented as mean ± SEM from 3 independent experiments performed in duplicate wells.
